# Why Should We Care About Molecular Coevolution?

**Published:** 2008-02-14

**Authors:** Francisco M. Codoñer, Mario A. Fares

**Affiliations:** 1 Evolutionary Genetics and Bioinformatics Laboratory, Department of Genetics, Smurfit Institute of Genetics, University of Dublin, Trinity College; 2 Institute of Immunology, Biology Department, National University of Ireland Maynooth

**Keywords:** Molecular coevolution, Mutual Information Content, parametric methods, non-parametric methods, protein-protein interactions

## Abstract

Non-independent evolution of amino acid sites has become a noticeable limitation of most methods aimed at identifying selective constraints at functionally important amino acid sites or protein regions. The need for a generalised framework to account for non-independence of amino acid sites has fuelled the design and development of new mathematical models and computational tools centred on resolving this problem. Molecular coevolution is one of the most active areas of research, with an increasing rate of new models and methods being developed everyday. Both parametric and non-parametric methods have been developed to account for correlated variability of amino acid sites. These methods have been utilised for detecting phylogenetic, functional and structural coevolution as well as to identify surfaces of amino acid sites involved in protein-protein interactions. Here we discuss and briefly describe these methods, and identify their advantages and limitations.

## Introduction

Revealing intra-molecular coevolution between amino acid sites of genes or gene regions has been one of the most important goals of genetists, bioinformaticians, experimentalists and of new emerging areas of research. Many methods have been devised to understand the evolutionary dynamic of organisms through the examination of multiple sequence alignments (MSA’s). Although this approach has dramatically improved our understanding of the mutational dynamics of proteins, the complexity of proteins’ mutability is beyond methods focusing on the analysis of linear sequences. The last decade has witnessed the emergence of a plethora of mathematical methods and computational tools aimed at drawing the spatial, functional and evolutionary dependencies between amino acid sites within a protein. The coevolutionary relationships between amino acid sites are however swamped in a background of different interacting factors governing the amino acids evolutionary dependency. During the last few years many efforts have been devoted to uncover coevolutionary relationships between amino acid sites belonging to the same or different proteins. The importance of such studies has been underpinned by many examples where dependencies between amino acid sites have unearthed the functional importance of residues (For example, see Fares and Travers, 2006; Travers and Fares, 2007).

The intrinsic complexity of the evolutionary dependencies between amino acid sites has however hampered the development of sensitive methods to detect functional coevolution. In fact coevolution between two amino acid sites can be decomposed into stochastic coevolution, functional coevolution and interaction coevolution. Each of these factors has different weights depending on, among other factors, how realistic models are to detect coevolution and the quality of the multiple sequence alignment. The sensitivity of most of parametric and non-parametric methods to detect functional coevolution (hereon, functional will refer to all those types of coevolution that do not involve stochastic or phylogenetic components) has been always compromised by the ability of these methods to disentangle the different types of coevolution.

As a result of the many challenges that detecting real coevolutionary dependencies between sites offers, many methods have developed trying to optimise the sensitivity and specificity to distinguish between the different types of evolutionary dependencies between amino acid sites.

In the light of the neutral theory of molecular evolution (Kimura, 1968) molecular changes are selectively neutral and therefore fixed by genetic drift (Hughes and Nei, 1988). This hypothesis implies that the fixation rate of mutations is constant throughout the evolutionary time (Bastolla et al. 2003), which is tantamount to the homogeneous distribution of substitution rates through generations. This theory has been challenged by several studies, with those relating the change in the rate of neutral mutations and protein’s structure being among the most interesting reports (Bastolla Vendruscolo and Knapp, 2000). These studies suggest that the fixation rate of amino acid substitutions depend upon more complex parameters that preclude the independence between amino acid sites as a possibility to explain molecular evolution.

## Inter-Dependent Evolution of Amino Acid Sites

The main reason for the amino acid sites interdependence is that proteins’ functions rely on their three-dimentional (3D) structure that relies on their complex functional and structural interaction networks. Identification of functionally or/and structurally related amino acid sites in a protein could shed light on the complex mutational dynamics that took place during the evolution of proteins. Functionally related amino acid residues are tightly evolutionarily linked because mutations at one position may very likely have dramatic effects on the dependent amino acid positions. Due to this dependency, the selection coefficient against changes in one amino acid site may be highly correlated with the complexity of its intra-molecular interaction networks. For any mutation hence to become fixed at such sites, compensatory mutations are needed at the related sites. This generates a dynamic of coevolution between functionally related sites and this dynamic has been regarded as an important phenomenon to understand processes of protein evolution.

**Figure 1:**
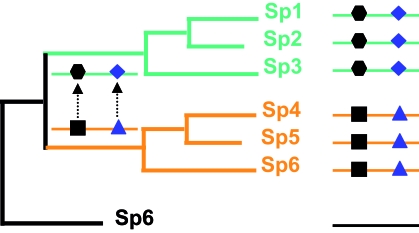
Phylogenetic coevolution. As mutations are fixed elsewhere in the sequence throughout the evolutionary time (black square mutating to a black hexagon), selective constraints on invariable regions may change (Triangle mutating to rhomboid).

This idea of coevolution relies on the idea of co-variation proposed by Fitch and Markowitz in the 70’s (Fitch and Markowitz, 1970), in which, in a particular time throughout evolution one region of the protein is invariable (due to structural or the functional constraints) while others accumulate mutations. As mutations are fixed elsewhere in the sequence throughout the evolutionary time, selective constraints on invariable regions may change (Figure. 1). Fitch later completed this concept of co-dependence between amino acid sites or protein regions (Fitch, 1971). This idea is essential in unveiling the mechanisms of molecular evolution, and might have pragmatic consequence for the structure prediction and drug design (Fares, 2006). Before its application to proteins, several authors have used the concept of coevolution to describe covariation between morphological characters (Pagel, 1994) or using DNA/RNA sequences (Schoniger and von Haeseler, 1994; Rzhetsky, 1995).

Detecting coevolving amino acid sites has been regarded as a good strategy for; (i) functional annotations of proteins encoded by unknown genes; (ii) revealing possible interactions between amino acids in the same protein; (iii) predicting protein-protein interactions; and (iv) understand how complex machineries undergo adaptive changes without having meaningful effects on the organism (Fraser et al. 2004).

## Detecting Molecular Coevolution

Due to the significant gap between available protein sequences and crystal structure for proteins, most of the studies on protein evolution are performed over the linear sequence. Conclusions drawn from these studies are incomplete because they ignore the third dimension (spatial dimension) that accounts for the dependence between linearly distant amino acid sites. Coevolution methods aimed at predicting atomic interactions between amino acids in a protein perform a powerful tool to unravel amino acid site dependencies ameliorating thus the problem of the lack of three-dimensional information (Göbel et al. 1994; Pazos et al. 1997). Because of the fact that functionally important amino acid regions in a protein are usually conserved throughout evolution, authors have focused their efforts on trying to identify such regions by conducting directed mutagenesis experiments (Sander and Schneider, 1993; Mirni and Shakhnovich, 1999; Wood and Pearson, 1999; Friedberg and Margalit, 2002; Oliveira et al. 2002; Oliveira et al. 2003; Nimrod et al. 2005). However, the number of possible experiments necessary to identify functional dependencies between amino acid sites overwhelmed the experimental capacities of most if not all the laboratories. To overcome these experimental limitations, many authors devised statistical methods and computational tools to identify functional dependencies between amino acid sites using intra-molecular coevolution analyses (for example, Fares and Travers, 2006; Travers and Fares, 2007).

Several parametric and non-parametric methods have been implemented to identify important residues in proteins. These methods focus on variable amino acid sites that can be functionally important, or that surround important functional domains and which covariation have a compensatory effect that maintains the three-dimensional structure characteristics (Taylor and Hatrick, 1994; Atwell et al. 1997; Chelvanayagam et al. 1997; Pazos et al. 1997; Olivera et al. 2002; Martin et al. 2005; Codoñer et al. 2006; Kim et al. 2006). Other methods have mostly focused on the detection of interaction between motifs or between proteins (Goh et al. 2000; Pazos and Valencia, 2001; Goh and Cohen, 2002; Pazos and Valencia, 2002; Ramani and Marcotte, 2003; Deng et al. 2006; Jothi et al. 2006; Kim and Subramaniam, 2006) or on the definition of protein-protein interaction networks (interactome) (Ju et al. 2003; Kim et al. 2004; Pazos et al. 2005; Chen and Yuan, 2006; Yu and Gerstein, 2006). Coevolution has been also instrumental for the in silico inference of the protein three-dimensional structure and resolution of docking problems (Göbel et al. 1994; Pazos et al. 1997).

New emerging parametric and non-parametric methods have devoted great part of their efforts on developing strategies to identify the components of coevolution.

The covariance between amino acid *i* and *j* in an alignment can be decomposed into:

$$C_{ij}=C_{phy\text{log}eny}+C_{Structure}+C_{function}+C_{\text{int}eractions}+C_{Stochastic}$$

**Figure 2:**
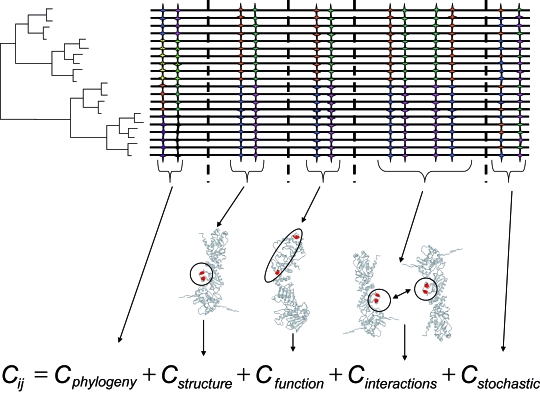
Decomposition of coevolution. Coevolution between two amino acid sites (*C_ij_*) can be decomposed into phylogenetic coevolution (*C_phylogeny_*), structural coevolution (*C_structure_*), functional coevolution (*C_function_*), coevolution due to atomic interaction (*C_interaction_*) and stochastic coevolution (*C_stochastic_*). Sites examined for coevolution are highlighted as colour stars in the multiple sequence alignment (group of horizontal lines). Dashed vertical lines separate different coevolutionary components. The different sequences (horizontal lines) are phylogenetically related following the topology shown.

*C_structure_* and *C_function_* account for co-variation due to the same selective forces acting on both sites to maintain a structural or functional domain (Atchley et al. 2000; Fig. 2). Phylogenetic covariation (*C_phylogeny_*) was exposed by Felsenstein (1985) to highlight the historical dependency between species and can hence be used for amino acid sites. On the other hand, among the remaining components of coevolution, *C_structure_* and *C_function_* are always very difficult to distinguish because they are not mutually exclusive and a pair of amino acid sites can be coevolving due to combination of different dependencies. Finally, *C_interactions_* usually reflects a functional and/or structural component, which make its distinction a rather difficult task. This component also implies that certain variation at the sequence level exists at amino acid sites involved in the interaction between two proteins. Even though interaction between amino acid sites implies coevolution between them, coevolution between sites does not necessarily mean they interact. Therefore, distinguishing coevolution due to interaction is easily mixed with that due to other factors. Stochastic covariation (*C_stochastic_*) can be due to convergent covariation of two sites due to the mutational dynamic of the sites (for example, accelerated fixation rates of evolution leading to saturation of amino acid sites). Removing the component of stochastic coevolution is more limited by our ability to model the dynamic of fixation of mutations at amino acid sites. Due to our inability to produce an analytical model to account for stochastic covariation, most methods rely on simulations of (MSAs). These MSAs share the same evolutionary parameters as the real MSA and can be used to produce a distribution of the probabilities to identify coevolution under a certain amino acid substitution model. Identifying stochastic coevolution is very much conditioned by the statistical properties of MSAs. Low-quality and poorly populated MSAs are more prone to produce false functional coevolution as a result of the significant effect of stochasticity on the detection of coevolution (Fares and Travers, 2006).

We consider two entities to coevolve when selective pressures in one specific entity drives the evolution of another entity (specificity) and, when this evolution happens, it occurs in both entities and at the same time (reciprocity and simultaneity) (Janzen, 1980). The entities under coevolution go from nucleotides, to amino acids, to proteins, to cells and even organisms. In this review we describe the most important methods to detect molecular coevolution and future directions in the identification of coevolution.

## Distance Matrix-Based Methods of Finding Correlation

**Figure 3:**
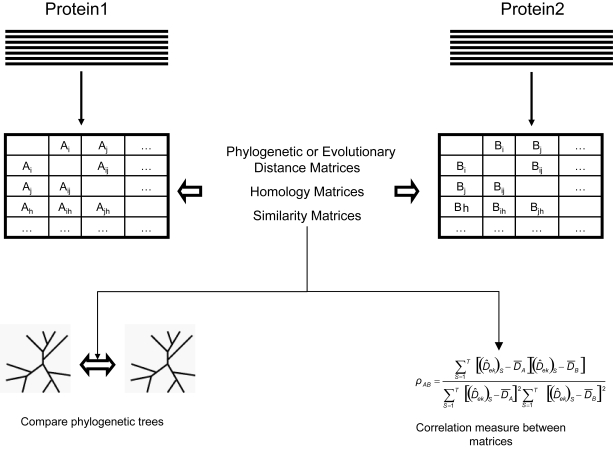
Algorithm diagram for coevolution methods based on the correlation of distance matrices. Multiple sequence alignments are used to estimate different kind of distance matrices, which are compared afterwards. A_i_ and B_i_ symbolise either the distance between two amino acid sites within the multiple sequence alignment or the distance between two proteins. The correlation between matrices together with the phylogenetic congruence are used to test coevolution between amino acid sites or proteins.

Several authors have used phylogenetic approaches to test the parallel evolution of interacting proteins. Adopting an inverse rationale, these authors have used the similarity in the phylogenetic branching order of proteins under study as an indicator or likelihood of their possible interaction (Moyle et al. 1994; Fryxell, 1996; van Kesteren et al. 1996; Yi et al. 2002). The correlation between the phylogenetic patterns of any two proteins can be used to estimate the probability of interaction between proteins (Pellegrini et al. 1999; Date and Marcotte, 2003). To test the phylogenetic correlation between interacting proteins we can compare tree distance matrices for the proteins under study. Several correlation measures have been developed during the last years to test the interaction between proteins. These methods were based on the correlation of the phylogentic or evolutionary distance matrices (Goh et al. 2000; Goh and Cohen, 2002; Kim et al. 2004; Pazos et al. 2005; Waddell, Kishino and Ota, 2006), on amino acid homology matrices (Göbel et al. 1994; Pazos et al. 1997; Pazos and Valencia, 2001; Pazos and Valencia, 2002; Hamilton et al. 2004), or on similarity matrices (Jothi et al. 2006). Also, we can use the correlation of the pattern of the presence of particular amino acid patterns in position *i* and *j* in a MSA as an indication of intra-molecular coevolution (Neher, 1994). The flow of the algorithm to detect coevolution for the methods explained above is depicted in Figure 3. Based on these approximations, authors have used evolutionary covariation between proteins to identify ligands-receptors interactions or to identify proximal coevolving amino acids in a three-dimensional structure (Pazos et al. 1997; Pazos and Valencia, 2001).

Among the main limitations of these methods, are the sizes of MSAs used and the background coevolution noise (Martin et al. 2005; Fares and Travers, 2006), and their inability to distinguish between positive and negative correlation (Pollock and Taylor, 1997). Several attempts have been made to reduce the background noise effect on the identification of functional coevolution, with the method of Neher (1994) being among the best ones. Among the possible reasons for the superiority of the method of Neher is the fact that this method weight the correlation coefficients between amino acid sites using a scalar metric based on the charge and volume of the side chains of the amino acids involved. Therefore, method of Neher does take into account biologically relevant information. Kim and colleagues (2004) also focused on studying the main limitations of correlation based methods to detect coevolution. They reported several causes for the low sensitivity of these methods to detect coevolution and protein-protein interaction, including among others (i) low correlation due to low diversity between the sequences in a MSAs of proteins belonging to the same family (Also see Pazos and Valencia, 2001); and (ii) low quality of the MSA.

## Non-Parametric Methods

The most extensively used methods to detect coevolution are those relying on non-parametric methods based on the Mutual Information Content (MIC). This approach is taken from the information theory (Kullback, 1959; Blahut, 1987; Farber et al. 1992), and is based on the variability that can be found in a protein alignment position. A formal measure of this variability is the Shannon entropy (*H_i_*) that is defined in terms of probabilities of the different symbols that can appear in the position i in the alignment, with these symbols corresponding to the 20 different amino acids. *H_i_* is defined as:

$$H(i)=-\sum_{s=A,S,L...}P(x_{i})\text{log}P(x_{i})$$

MIC definition involves the joint probability distribution, *P*(*x_i_*, *y_j_*), of the occurrence of symbol *x* at position *i* and symbol *y* at position *j* belonging to the same protein or to two different proteins.

$$H(i,j)=-\sum_{x_{i},y_{j}}P(x_{i},y_{j})\text{log}P(x_{i},y_{j})$$

So *MI* values is calculated as:

MI=H(i)+H(j)-H(i,j)

where *H*(*i*) is the measure of the variability at the amino acid site *i*, *H*(*j*) the measure of the variability at site *j* and *H*(*i,j*) is the join probability as described above. *MI* values range between 0, indicating independent evolution between sites *i* and *j* or conserved amino acid sites, and a positive value whose magnitude depends on the strength of covariation between sites (Blahut, 1987). Further, the power of *MI* to predict real coevolution is highly dependent on the level of background conservation in the MSA, as highlighted in a study testing the influence of conservation on calculations of covariance between amino acid sites in MSA (Fodor and Aldrich, 2004).

The advantage of using *MI* values to quantify the amount of coevolution, and thus the probability of interaction between proteins or amino acids, relies on the applicability of the method without knowledge about the relationship between the residues in the MSA or the evolutionary dynamic of these residues. Because of the historical dependence between amino acid sites, *MI* values are always affected by these dependencies unless otherwise corrected explicitly in the model to detect coevolution. Several studies have attempted to address the correction of *MI* values by subtracting the effect of phylogenetic dependence between amino acid sites (Atchley et al. 2000; Wollenberg and Atchley, 2000; Tillier and Lui, 2003; Buck and Atchely, 2005). However, most of these methods applied adhoc corrections to the problem resulting in a decrease in their sensitivity to detect true coevolutionary relationships. Further, many of the studies using MIC to detect coevolution, focused on detecting specific coevolving amino acid sites within a protein or protein motif as a strategy to ameliorate the problem of false positive results in the analyses (Korber et al. 1993; Clarke, 1995; Atchely et al. 2000; Wollenberg and Atchley, 2000; Olivera et al. 2002; Hoffman et al. 2003; Tillier and Lui, 2003; Weckwerth and Selbig, 2003; Buck and Atchley, 2005; Gloor et al. 2005; Hummel et al. 2005). Many case studies have yielded important information regarding the coevolution in active sites or regions surrounding important functional domains in proteins (Olivera et al. 2002; Weckwerth and Selbig, 2003; Gloor et al. 2005). These methods have been also used to reveal gene functional annotations using MIC profiles in combination with other approximations (Zeng et al. 2002). The rationale behind this approach is that genes showing similar functions do normally coevolve. Under this assumption, genes sharing the same coevolution pattern across different genomes are expected to have related functions. Consequently, protein functions can be inferred using coevolutionary analyses when comparing with proteins already annotated.

Coevolution between proteins known to interact has been also used to identify amino acid sites involved in protein-protein interactions (Martin et al. 2005; Codoñer et al. 2006; Tillier et al. 2006). Authors have also used coevolution analyses based on MIC to identified protein-protein interactions by comparing phylogenetic profiles between proteins or domains (Kim et al. 2006; Kim and Subramaniam, 2006), by indirectly measuring networks of gene interactions (Chen et al. 2006), or in combination with other methods to detect differences in the amount of functional and structural sites between transient and permanent protein-protein interactions (Mintseris and Weng, 2005). These approximations are very useful when the purpose is to highlight specific amino acid sites in the protein responsible for the interaction between residues.

As we mention elsewhere in the manuscript, sensitivity of most of the methods developed to detect coevolution using MI values depends on (i) the reliability of the MSA, (ii) the number of sequences in the MSAs; and (iii) the mean pairwise divergence levels in the MSAs.

## Parametric Methods

Parametric approximations have not received much attention in comparison with non-parametric methods. The main reason is that parametric methods are developed around variables that are based on several assumptions. These methods are therefore subjected to several inaccuracies prompted by our limited knowledge of the process of between-residues evolutionary interaction and therefore by the simplistic assumptions made by the models. Assuming these limitations, authors have developed several models to detect coevolution using formally developed probabilistic models, based on maximum likelihood approximation (Pollock, Taylor and Goldman, 1999; Choi, Li and Lahn, 2005; Pei et al. 2006), on Bayesian probabilities (Dimmic et al. 2005), on phylogentic approaches (Fukami-Kobayashi et al. 2002), or on sequence divergence based approximation (Fares and Travers, 2006; Fares and McNally, 2006).

These methods have also incorporated several correction measures to account for the noise caused by the non-independence between sequences. For example, some methods have implemented accurate inferences of ancestral sequences (Pollock, Taylor and Goldman, 1999). Although, these methods improved the sensitivity to detect coevolution under specific datasets, they showed limited sensitivity to identify real coevolution. For example, the method developed by Pollock and colleagues makes the simplistic assumption of constant coevolutionary relationships between amino acid sites and is limited to closely related protein families (Pollock, Taylor, and Goldman, 1999).

Choi and colleagues (2005) used the increment in the log-likelihood values for the phylogeny of orthologous proteins to detect coevolving positions. Other methods have used the phylogentic information to identify compensatory mutations in MSAs (Fukami-Kobayashi et al. 2002).

In contrast to the phylogenetic approaches, the method developed by Fares and Travers (Fares and Travers, 2006; Fares and McNally, 2006) is capable of distinguishing between background and true correlations with no knowledge about the phylogenetic relationships between sequences. This method corrects pairwise sequence divergence values by the strength of the amino acid substitutions using BLOcks Substitutions Matrix 62 (BLOSUM62) values. Then correlation of divergence values between amino acid sites is estimated to identify significant coevolutionary relationships between amino acid sites. In addition, it includes three-dimensional information to identify functional and structural pairs of coevolving sites. This method is also subjected to several limitations among which are important the saturation of synonymous sites; low number of sequences in the MSA; High pairwise divergence levels and inability to identify conserved coevolving sites.

Despite the many limitations of parametric methods, these have been regarded as presenting more statistical power than non-parametric methods (Pollock, Taylor and Goldman, 1999; Fukami-Kobayashi et al. 2002; Dimmic et al. 2005; Fares and Travers, 2006; Pei et al. 2006). These methods have been also shown to present greater sensitivity to detect coevolving residues sharing weak signal of coevolution.

## Other Methods

Gene expression correlation between interacting proteins has been also used as a measure of coevolution (Fraser et al. 2004). Rather than using covariation between amino acid sites, this method used co-expression between proteins as a measure of coevolution. The rationale behind this method is that correlation in the expression levels of two proteins is more likely to account for the interaction between the proteins because interacting proteins have to present similar abundances in the cell. Authors thus regarded this measure as being more powerful in detecting coevolution than conventional covariation based methods (Fraser et al. 2004). Further, authors have highlighted the goodness-of-fit of this method in comparison to methods based on phylogenetic profiles (Pellegrini et al. 1999) or conservation of gene neighbourhood (Dandekar et al. 1998), because it is not limited by the presence or absence of genes in different species or by the information of syntheny in other related species.

Another method used for detecting protein coevolution is the one developed by Ramani and Marcotte (2003). This method compares trees inferred for ligands and their receptors, and creates distance matrices for both alignments based on their phylogenetic trees. The method fixes then one of the matrices and shuffles rows and columns in the other distance matrix as to maximize the number of coincidences and minimise the root mean square difference between the elements of the two matrices. Interacting proteins will be those that have equivalent columns. They also use the three-dimensional based information to visualize the interacting partners, and estimate the MI values to infer the accuracy of the method. Pritchard and co-workers (2001) also developed a method based on finding patterns of amino acids in specific positions of the MSAs being compared. This method looks for correlated variation between two amino acid sites by splitting the patterns of amino acid pairings between the sites into defined blocks. A pair of amino acids (A and B) defines each block and the occurrence of these amino acids is restricted to that particular block. The number of sequences in these blocks (size of the blocks) and the frequencies of the amino acids are used in the estimation of the correlated variation of the two sites examined. Pritchard and colleagues tested the accuracy of the method using several simulated datasets and showed that the sensitivity of the method is greater than that of other non-parametric and parametric methods. Among the greatest advantages was the fact that the number of sequences at which sensitivities were acceptable was low (around 16 sequences). However sensitivity is greatly dependent on the level of amino acid variability in the MSA. Another assumption made by the authors was that there are no shifts on the pairings of amino acids throughout evolution. The same pairs coevolve throughout the evolutionary time of the species. This test then can very likely fail when dealing with paralogous sequences, where the shift in the evolutionary constraints are very probable after the gene duplication.

## Future Challenges

Most of the methods exposed in this review have the limitation of being highly dependent on the quality of the MSAs regarding the number of sequences, the mean pairwise sequence divergence levels as well as the amount of sequence variability information contained on the different amino acid sites. Future work should be focusing on minimising the effects of these factors on the sensitivity of the different parametric and non-parametric methods to detect coevolution. For example, introducing models capable of accurately quantifying and detecting stochastic amino acid sites covariation would be desirable especially when the number of sequences or the amount of biological information are limited. More work is also needed on improving the ability of methods to detect protein-protein interfaces and to disentangle functional coevolution from stochastic and phylogenetic coevolution. Regarding parametric methods to detect coevolution, introducing parameters accounting for biological information will lead to more realistic models that will tackle the problem of stochastic covariation.
